# Stiffness-Based Evaluation of Hinge Joints in Prefabricated Assembled Multi-Girder Bridges under Operational Conditions

**DOI:** 10.3390/s24134255

**Published:** 2024-06-30

**Authors:** Zhiqiang Shang, Fengzong Gong, Zhufeng Shao, Jose Matos, Gongfeng Xin, Ye Xia

**Affiliations:** 1 Shandong Hi-Speed Group Innovation Research Institute, Jinan 250102, China; 2 Shandong Key Laboratory of Highway Technology and Safety Assessment, Jinan 250102, China; 3 Department of Bridge Engineering, Tongji University, Shanghai 200092, China; 4 Shandong Hi-Speed Engineering Testing Co., Ltd., Jinan 250100, China; 5 Department of Civil & Environmental Engineering, University of Minho, 4710-057 Braga, Portugal

**Keywords:** prefabricated assembled multi-girder bridge, hinge joint, damage identification, modal analysis

## Abstract

Presently, the prevailing approaches to assessing hinge joint damage predominantly rely on predefined damage indicators or updating finite element models (FEMs). However, these methods possess certain limitations. The damage indicator method requires high-quality monitoring data and demonstrates variable sensitivities of distinct indicators to damage. On the other hand, the FEM approach mandates a convoluted FEM update procedure. Hinge joint damage represents a major kind of defect in prefabricated assembled multi-girder bridges (AMGBs). Therefore, effective damage detection methods are imperative to identify the damage state of hinge joints. To this end, a stiffness-based method for the performance evaluation of hinge joints of AMGBs is proposed in this paper. The proposed method estimates hinge joint stiffness by solving the characteristic equations of the multi-beam system. In addition, this study introduces a method for determining baseline joint stiffness using design data and FEM. Subsequently, a comprehensive evaluation framework for hinge joints is formulated, coupling a finite element model with the baseline stiffness, thereby introducing a damage indicator rooted in stiffness ratios. To verify the effectiveness of the proposed method, strain and displacement correlations are analyzed using actual bridge monitoring data, and articulation joint stiffness is identified. The results underscore the capability of the proposed method to accurately pinpoint the location and extent of hinge joint damage.

## 1. Introduction

Prefabricated assembled multi-girder bridges (AMGBs) stand out as a prominent choice for small and medium-sized span bridges. In this bridge type, a horizontal arrangement of prefabricated slab beams is prevalent, and these beams are interconnected by hinge joints. These hinge joints collectively establish the foundational load-bearing structure of the bridge featuring hinged slab beams. Notably, hinge joints fulfill the dual role of load transmission and structural system integration. However, they simultaneously constitute vulnerable points within the broader load-bearing framework [1]. Hinge joint damage represents a frequently encountered form of deterioration in hinged plate beam bridges. Such damage has the potential to induce alterations in the distribution of forces across the prefabricated beams, thereby exerting an impact on the overall safety and long-term resilience of the bridge structure.

Within the realm of bridge inspection, the assessment of hinge joint conditions can be accomplished through visual inspection. Hinge joint damage usually manifests itself in a variety of indicators, such as bridge deck rutting, seepage at joints, and the misalignment of main girders [2,3,4,5]. By detecting the characteristics of these issues, hinge joints can be evaluated. Nevertheless, it is important to acknowledge that visual inspection primarily exposes overt defects within the bridge structure. As the field of bridge health monitoring technology progresses, an ever-growing array of bridges are being equipped with health monitoring systems. The primary objective of implementing these systems is to gauge the state of bridges by dissecting the data gleaned from the monitoring mechanisms in place [6]. In the health monitoring research of hinged plate beam bridges, two typical methods are commonly employed to evaluate the state of hinge joints [7]: the damage indicator method and the finite element model (FEM) updating method.

The damage indicator method relies on monitoring indicators to assess the structural condition, with the most commonly utilized being a category of correlation indicators, including strain [8], response spectrum [9,10], modal shapes [11], and load lateral distribution coefficient [12]. Han et al. [13] proposed a vibration-based damage index for hinge joints. Hu et al. [14] employed a computer vision approach to monitor the dynamic displacement of a bridge and then evaluated the articulation joints based on displacement-related metrics. Dan et al. [9,11,15,16] proposed a series of correlation indicators, including modal shapes, strain, and displacement. Xia [17] suggested utilizing the load lateral distribution factor to evaluate hinge joints. Walsh [18] explored a new damage detection method based on the first vertical mode change extracted from the lateral movement of a bridge. Although some methods are usually relatively simple and fast in calculation, these indirect damage indicators are not intuitive and require the establishment of corresponding relationships between indicators and damage. Furthermore, some indicators have non-linear relationships with damage [14], indicating that the sensitivity of the indicators varies for different levels of damage.

The FEM updating method involves establishing an FEM of a bridge, combining it with actual monitoring data, integrating it with actual monitoring data, employing numerical optimization algorithms to determine the stiffness value of the hinge joint, and evaluating the damage status of the hinge joint. Abedin [19] conducted load tests on a precast box girder bridge that had been in service for over 50 years and evaluated the bridge performance using an FEM updating method. Zhan [20] proposed a method combining vehicle–bridge coupling theory and FEM updating to assess damage to bridge hinge joints. Although the method based on FEM updating can directly obtain the stiffness of hinge joints, this method requires the establishment of an accurate FEM. The complex FEM updating process significantly increases the workload and calculation time for hinge joint evaluation.

This study developed a method for identifying the stiffness of hinge joints to propose a hinge joint evaluation framework. First, modal shapes were identified using monitoring data, and the stiffness of hinge joints was determined by solving the characteristic equations of the multi-beam system. Evaluating the condition of hinge joints requires not only the actual stiffness values of each hinge joint but also a baseline value to determine which hinge joints are damaged. In practical engineering applications, it is a challenge to determine the baseline stiffness of a bridge that has not undergone load testing. Therefore, this study presents a method for determining the baseline stiffness of articulated joints based on design information and FEM. This study developed a comprehensive framework for joint assessment. Finally, the evaluation framework utilized FEM to derive a method for calculating the stiffness baseline value and proposed a damage indicator based on the stiffness ratio. The proposed method in this paper not only enables the convenient identification of damaged hinge joints using the stiffness ratio metric but also provides a quantitative measure of the stiffness of each hinge joint.

The remaining part of this article is organized as follows: Section 2 proposes an identification method for stiffness and a framework for evaluating hinge joints. The accuracy of the proposed method was verified through numerical simulation, discussed in Section 3. The proposed method was validated on an actual bridge and compared with strain and displacement correlation indicator methods, discussed in Section 4. Section 5 summarizes the conclusions.

The main contributions of this paper are as follows: (1) A comprehensive framework for the assessment of hinge joints is proposed that not only yields the actual stiffness of joints but also gives the baseline value of the stiffness. The proposed stiffness ratio-based damage index is more practical. (2) The proposed method was validated on a real bridge. (3) The correlations of displacement and strain monitoring data are analyzed.

## 2. Hinge Joint Evaluation Method

### 2.1. Identification of Hinge Joint Stiffness

**Figure 1:**
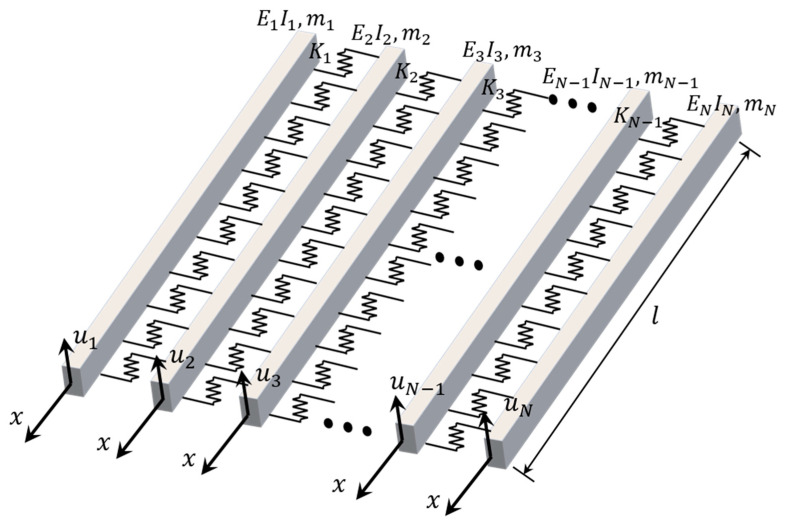
Multi-beam model.

This section proposes a method for identifying the stiffness of hinge joints. A multi-beam model was used as the mechanical model of the prefabricated AMGB, as shown in Figure 1. The following assumptions were made when simplifying the AMGB to a mechanical model:

1. Hinge joints transmit only shear forces and are considered as Winkler elastic layers;
2. The torsional and transverse displacements of the beams should be neglected;
3. Properties of beams and hinge joints along the length are uniform.

The governing differential equation for the free vibration of a multi-beam system is as follows [21]:

(1) $$E_{1}I_{1}\frac{\partial^{4}u_{1}\left(x,t\right)}{\partial x^{4}}+m_{1}\frac{\partial^{2}u_{1}\left(x,t\right)}{\partial t^{2}}+K_{1}\left(u_{1}\left(x,t\right)-u_{2}\left(x,t\right)\right)=0$$

(2) $$E_{n}I_{n}\frac{\partial^{4}u_{n}\left(x,t\right)}{\partial x^{4}}+m_{n}\frac{\partial^{2}u_{n}\left(x,t\right)}{\partial t^{2}}+K_{n}\left(u_{n}\left(x,t\right)-u_{n+1}\left(x,t\right)\right)+K_{n-1}\left(u_{n}\left(x,t\right)-u_{n-1}\left(x,t\right)\right)=0,\;\;\;n=2,3,\ldots ,N-1$$

(3) $$E_{N}I_{N}\frac{\partial^{4}u_{N}\left(x,t\right)}{\partial x^{4}}+m_{N}\frac{\partial^{2}u_{N}\left(x,t\right)}{\partial t^{2}}+K_{N-1}\left(u_{N}\left(x,t\right)-u_{N-1}\left(x,t\right)\right)=0$$

where $u_{n}\left(x,t\right)$ is the vertical displacement of the *n*th beam, $E_{n}I_{n}$ is the bending stiffness of the *n*th beam, $m_{n}$ is the mass of the *n*th beam, and $K_{n}$ is the stiffness of the hinge joint between the *n*th beam and the (*n +* 1)th beam.

The solution $u_{n}\left(x,t\right)$ is as follows:

(4) $$u_{n}\left(x,t\right)=\varphi_{n}\left(x\right)e^{i\omega t}$$

where $\varphi_{n}\left(x\right)=A_{n}e^{\kappa x}$ is the modal shape function and $A_{n}$ is the mode amplitude of the *n*th beam. By incorporating Equation (4) into Equations (1)–(3), the characteristic equation of the multi-beam system could be obtained as follows:

(5) $$\left[\begin{array}{ccccc} E_{1}I_{1}\kappa^{4}+K_{1}-m_{1}\omega_{j}^{2} & -K_{1} & 0 & \cdots & 0\\ -K_{1} & E_{2}I_{2}\kappa^{4}+K_{1}+K_{2}-m_{2}\omega_{j}^{2} & -K_{2} & \cdots & 0\\ \vdots & \vdots & \vdots & \ddots & \vdots \\ 0 & 0 & 0 & \cdots & E_{N}I_{N}\kappa^{4}+K_{N-1}-m_{N}\omega_{j}^{2} \end{array}\right]\left[\begin{array}{c} A_{1}\\ A_{2}\\ \vdots \\ A_{N} \end{array}\right]=\left[\begin{array}{c} 0\\ 0\\ \vdots \\ 0 \end{array}\right]$$

where the mass of the *n*th beam $m_{n}$ can be calculated based on the design parameters of the bridge. The vibration mode amplitude of the *n*th beam $A_{n}$ could be identified using vibration data measured by the installed accelerometers. Considering that the cross-section as well as the material parameters of all main beams were uniform, it could be assumed that the elastic modulus and moment of inertia of each main beam were equal. Given these parameters and assumptions, there were only *n* unknowns in the above equation, i.e., ${\left[EI\kappa^{4},\;K_{1},K_{2},\ldots ,\;K_{N-1}\right]}^{T}$. Therefore, the *n* equations in Equation (5) could be used to solve these *n* positional parameters, and Equation (5) could be rewritten as

(6) $$\left[\begin{array}{ccccc} E_{1}I_{1}\kappa^{4}A_{1}+K_{1}A_{1} & -K_{1}A_{2} & 0 & \cdots & 0\\ -K_{1}A_{1} & E_{2}I_{2}\kappa^{4}A_{2}+K_{1}A_{2}+K_{2}A_{2} & -K_{2}A_{3} & \cdots & 0\\ \vdots & \vdots & \vdots & \ddots & \vdots \\ 0 & 0 & 0 & \cdots & E_{N}I_{N}\kappa^{4}A_{N}+K_{N-1}A_{N} \end{array}\right]=\omega_{j}^{2}\left[\begin{array}{cccc} m_{1} & 0 & \cdots & 0\\ 0 & m_{2} & \cdots & 0\\ \vdots & \vdots & \ddots & 0\\ 0 & 0 & \cdots & m_{N} \end{array}\right]\left[\begin{array}{c} A_{1}\\ A_{2}\\ \vdots \\ A_{N} \end{array}\right]$$

(7) $$\left[\begin{array}{ccccc} A_{1} & A_{1}-A_{2} & 0 & \cdots & 0\\ A_{2} & -\left(A_{1}-A_{2}\right) & A_{2}-A_{3} & \cdots & 0\\ A_{3} & 0 & -\left(A_{2}-A_{3}\right) & \cdots & 0\\ \vdots & \vdots & \vdots & \ddots & \vdots \\ A_{N} & 0 & 0 & \cdots & -\left(A_{N-1}-A_{N}\right) \end{array}\right]\left[\begin{array}{c} EI\kappa^{4}\\ K_{1}\\ K_{2}\\ \vdots \\ K_{N-1} \end{array}\right]=\omega^{2}M_{n}A_{n}$$

where $M_{n}$ is the mass matrix and $A_{n}={\left[A_{1},A_{2},\ldots ,A_{N}\right]}^{T}$ is the modal shape vector.

By solving the above equations, the stiffness of the bridge hinge joint $K_{1},K_{2},\ldots ,K_{N-1}$ could be obtained. It is worth noting that the boundary conditions of the bridge were not specified during the derivation process, indicating that the proposed method is not only applicable to simply supported girder bridges but also to more complex boundary conditions. In practical bridge monitoring, multi-order bridge modes can usually be obtained, and, therefore, the obtained multi-order modes could be used to simultaneously solve Equation (7).

### 2.2. Estimation of Baseline Stiffness

After obtaining the measured stiffness of the hinge joints, it was essential to have the baseline stiffness for comparison to identify which hinge joints had experienced damage. In practical engineering, the actual stiffness in the undamaged state may be unknown. This section provides an FEM-based approach to estimate the baseline stiffness. A straightforward method is to select the maximum hinge joint stiffness calculated in Section 2.1 as the baseline. However, this approach results in the stiffness of all the other hinge joints being lower than the baseline, which may hinder the localization of possible damages.

The design information for the structure provided important reference information to help determine the articulation joint stiffness of the bridge under normal conditions. The initial joint stiffness of the bridge could be further determined by establishing the FEM of the bridge from the design information. However, in general, the dynamic characteristics of FEMs built based on design data usually deviate from those of actual bridges. An FEM that relies solely on design data may yield unreliable results. To address this, the FEM was first established according to design parameters, followed by an optimization process to update the stiffness of each beam and hinge joint. This was done by minimizing the discrepancy between the measured natural frequency and the calculated natural frequency of each vibration mode:

(8) $$K_{b}=argmin\left(\sum_{j=1}^{m}\left|f_{j}^{FEM}-f_{j}^{m}\right|\right)$$

where $k_{b}$ is the baseline of joint stiffness, $f_{j}^{FEM}$ is the *j*th frequency of the FEM, and $f_{j}^{m}$ is the *j*th measured frequency. During the optimization process, two assumptions were retained that were consistent with the design profile: 1. all girders have the same stiffness and 2. all articulated joints have the same baseline stiffness, since all the hinge joints were designed with uniform parameters and constructed using uniform materials. The assumption that all hinge joints had the same stiffness was adopted in establishing the benchmark FEM. It should be noted that if the bridge was already damaged, the baseline stiffness obtained from the monitoring data could be low. However, for screening out articulated joints with a high degree of damage, this baseline stiffness was still available.

As for numerical optimization algorithms, there has been a great deal of related research, such as that on the Genetic Algorithm (GA) [22,23] and Particle Swarm Optimization (PSO) [24,25]. The details of these algorithms are not covered in this paper and can be found in relevant references. In this paper, PSO was used to optimize the benchmark FEM. Specifically, the objective function $F\left(K_{i}\right)$ for optimizing the FEM was that according to Equation (8).

### 2.3. Hinge Joints Assessment Framework

**Figure 2:**
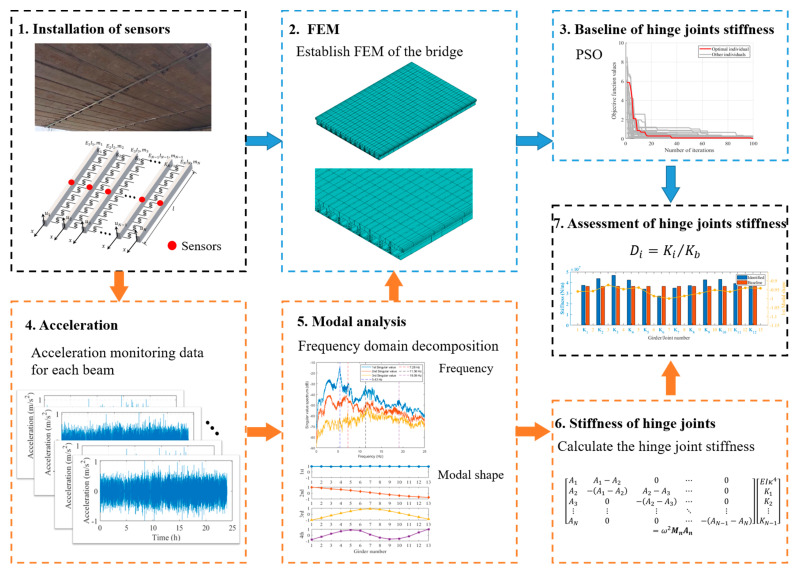
Hinge joint assessment framework.

Combining the identification method proposed in Section 2.1 with the baseline estimation methods in Section 2.2, a framework for evaluating hinge joint performance could be developed. As shown in Figure 2, the framework mainly included two steps:

Step 1: Hinge joint stiffness identification of real bridge (1-4-5-6)

The purpose of this step was to evaluate the stiffness of actual bridge hinge joints. First, acceleration sensors were installed at the mid-span of each girder in the bridge, such that the acceleration data of the bridge could be obtained. Then, the natural frequencies and modal shapes of the bridge were identified using the general Operational Modal Analysis (OMA) method. Finally, the stiffness of the hinge joint between each two girders was calculated using the stiffness identification method proposed in Section 2.1.

Typical OMA methods include Stochastic Subspace Identification (SSI) [26], the Eigensystem Realization Algorithm (ERA) [27], Frequency Domain Decomposition (FDD) [28], etc. These methods have been widely used in bridge modal identification in operational environments. In this paper, the FDD method, one of the most widely used OMA methods, was applied to identify the vibration modes of the real bridge. Compared with SSI and ERA, FDD avoids the problem of false modes related to determining the frequencies and modal shapes of the bridge.

Step 2: Baseline establishment for hinge joint performance evaluation (1-2-5-3-7)

The objective of this step was to establish the stiffness limit for hinge joints and provide corresponding damage indicators. A damage indicator was defined using baseline stiffness and the identified stiffness explained in Section 2.1, such that the performance evaluation of hinge joints could be achieved. Once the baseline stiffness was obtained, the damage indicator $D_{i}$ of i-th hinge joint could be defined as follows:

(9) $$D_{i}=\frac{K_{i}^{\prime }}{K_{b}}$$

where $K_{i}^{\prime }$ is the identified joint stiffness using the method proposed in Section 2.1 and $K_{b}$ is the baseline stiffness obtained by optimizing the benchmark FEM.

As the baseline stiffness used in Equation (8) was an estimated value rather than the actual stiffness in the undamaged state, $D_{i}$ was actually an approximation of the extent of hinge joint damage. When $D_{i}\;\geq \;1$, the identified stiffness was greater than the baseline, indicating that the hinge joint was in an undamaged state. Conversely, when $D_{i}<1$, the identified stiffness was lower than the baseline, suggesting that damage may have occurred in the corresponding hinge joint.

In summary, the proposed evaluation framework acquired the measured stiffness values of each hinge joint, allowing the determination of the location of damage through the predefined damage indicator. Similarly, the extent of damage could also be approximated using the same method.

## 3. Numerical Verification

### 3.1. Stiffness Identification

**Figure 3:**
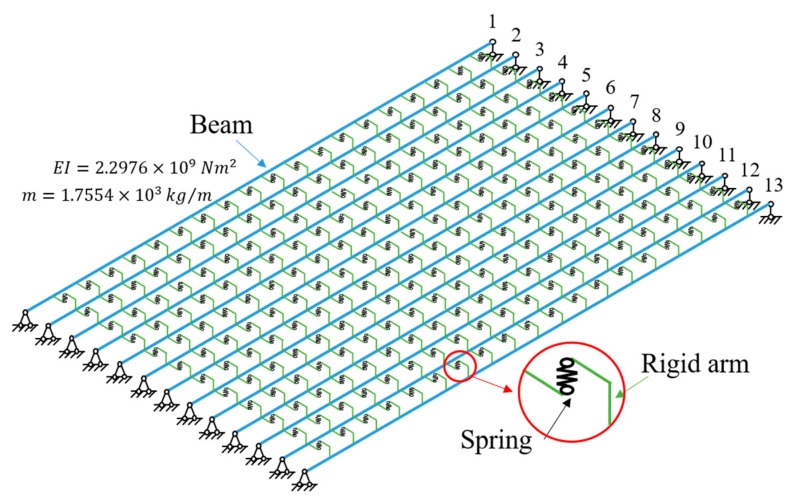
Model for numerical simulation.

In order to verify the accuracy of the method proposed in Section 2.1, this study established an FEM of the multi-beam system, calculated the dynamic response of the multi-beam system under white noise excitation, and then identified the stiffness of the hinge joint. The multi-beam system consisted of 13 identical beams, each with a length of 20 m, $EI=2.2976\times 10^{9}\;Nm^{2}$, $m=1.7554\times 10^{3}\;kg/m$, and a simply supported boundary condition. The stiffness of the hinge joint was $K=1\times 10^{7}\;N/m$. Using the Rayleigh damping matrix, the first-order damping ratio was set to 2%. The model graph is shown in Figure 3. During the discretization process of building the finite element model, care was taken to convert the continuous spring to discrete spring stiffness.

The following two damage conditions were set as follows:

Case 1: No. 4 hinge joint stiffness $0.4\times 10^{7}\;N/m$, No. 6 hinge joint stiffness $0.7\times 10^{7}\;N/m$;

Case 2: No. 3 hinge joint stiffness $0.8\times 10^{7}\;N/m$, No. 4 hinge joint stiffness $0.6\times 10^{7}\;N/m$, No. 8 hinge joint stiffness $0.8\times 10^{7}\;N/m$, No. 12 hinge joint stiffness $0.4\times 10^{7}\;N/m$.

The frequencies obtained from the bridge FEM calculations are shown in Table 1. When damage occurred in the hinge joints of the bridge, the frequencies of the first-order overall vertical bending did not change, while the torsional frequencies were all slightly reduced.

**Figure 4:**
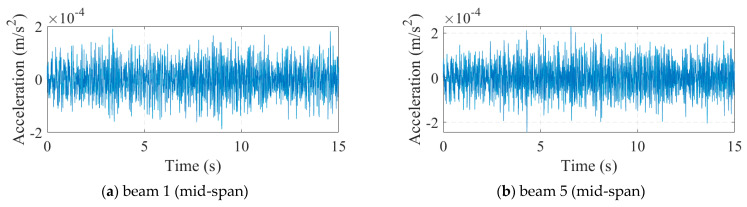
Acceleration of Case 1.

**Figure 5:**
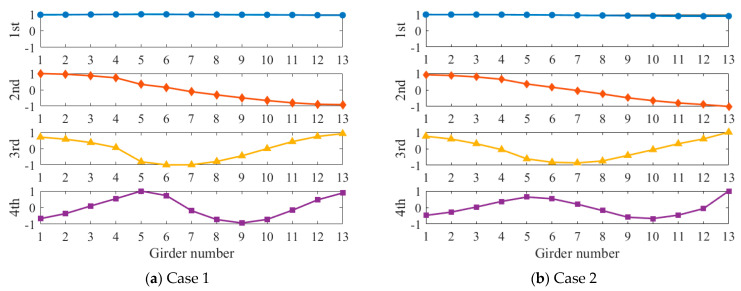
Identified modal shapes of Case 1 and Case 2.

**Figure 6:**
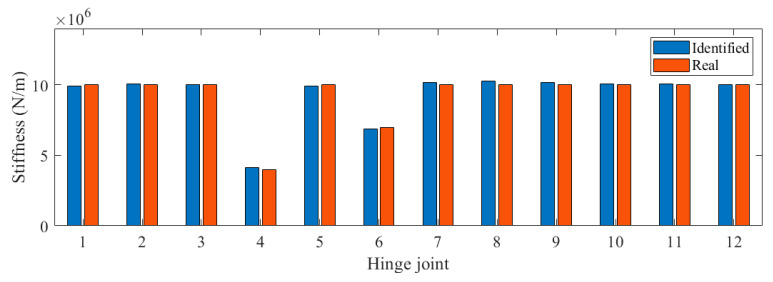
Identified stiffness in Case 1.

**Figure 7:**
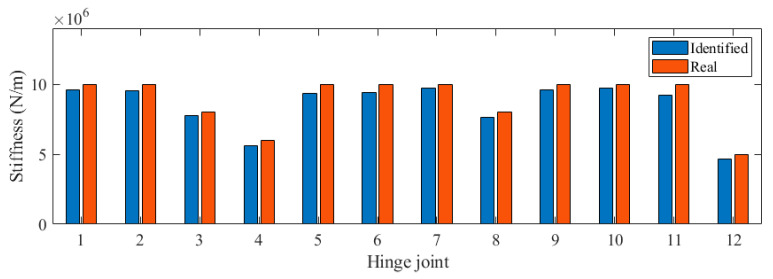
Identified stiffness in Case 2.

We applied white noise loads to each beam and used the Newmark method to calculate the dynamic response of the bridge, with a calculation time step of 0.005 s. We obtained the acceleration response of each main beam span, as shown in Figure 4. It can be seen that there was no significant difference in acceleration. Then, the FDD method was applied to identify the vibration mode of the bridge, and the results are shown in Figure 5. The second and third modes of the bridge underwent changes at the position of the fourth hinge joint, but the changes at the position of the sixth hinge joint were not significant. It was found that based only on the bridge modes in the transverse direction, it was difficult to accurately evaluate the damage situation of the bridge hinge joint. We further used the stiffness identification method for hinge joints in Section 2.1, and the results are shown in Figure 6 and Figure 7. It can be seen that the proposed method could accurately identify the stiffness of each hinge joint.

### 3.2. Damage Indicator

**Figure 8:**
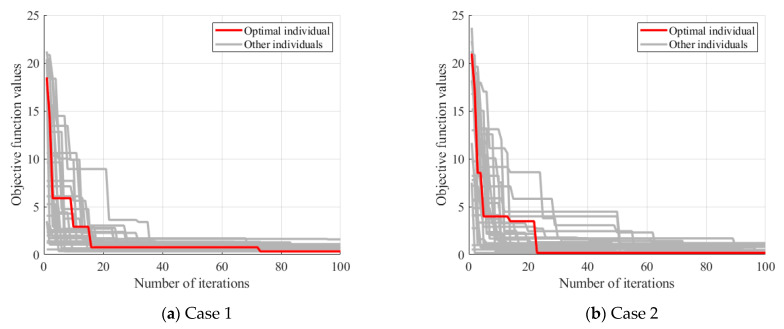
Baseline stiffness optimization process.

The baseline stiffness of Case 1 and Case 2 were further estimated using the method of Section 2.2. The baseline stiffness was optimized using the PSO algorithm and the optimization process is shown in Figure 8. When the number of iterations reached 100, the objective function value generally converged. The baseline stiffness for Case 1 and Case 2 were obtained as $0.93\times 10^{7}\;N/m$ and $0.83\times 10^{7}\;N/m$, respectively. The obtained baseline stiffness was slightly lower than that of the undamaged state. Nonetheless, it was still feasible to screen out hinge joints with a relatively large degree of loss using the baseline stiffness.

The damage indicators were calculated and are shown in Table 2. In Case 1, the damage indexes of all undamaged joints were greater than 1, while the damage indexes of damaged joint 4 and joint 6 were 0.44 and 0.73, respectively. This indicated that the damage indexes proposed in this paper could successfully differentiate the damage states, and the values of the damage indexes were basically the same as the extent of hinge joint damage. In Case 2, the proposed damage metrics also correctly distinguished the damage state of each hinge joint. The results of the numerical simulations showed the validity of the proposed assessment framework, based on which, the damage of hinge joints could be quantitatively assessed.

## 4. Application on a Real Bridge

### 4.1. Bridge Description

**Figure 9:**
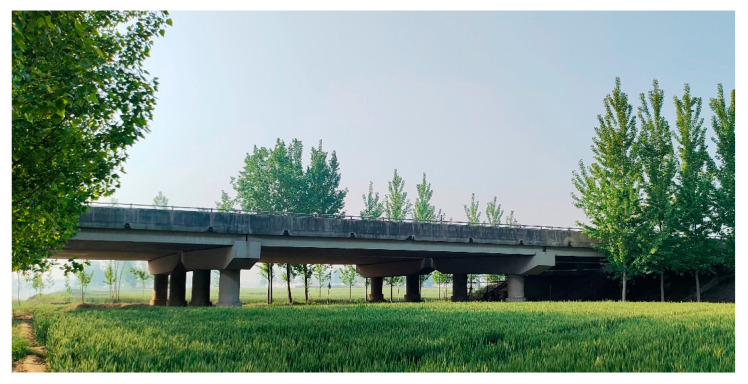
Side view of the bridge.

**Figure 10:**
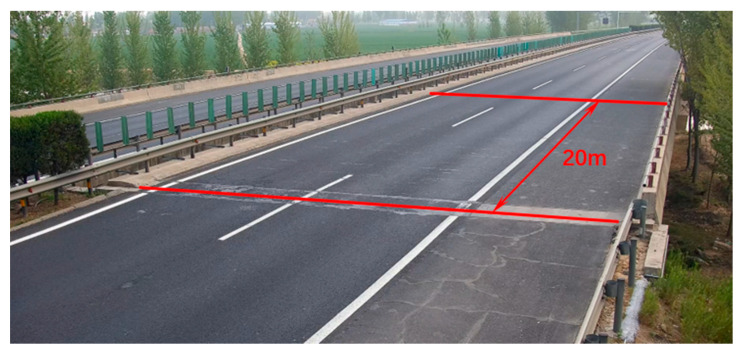
Top view of the bridge.

**Figure 11:**
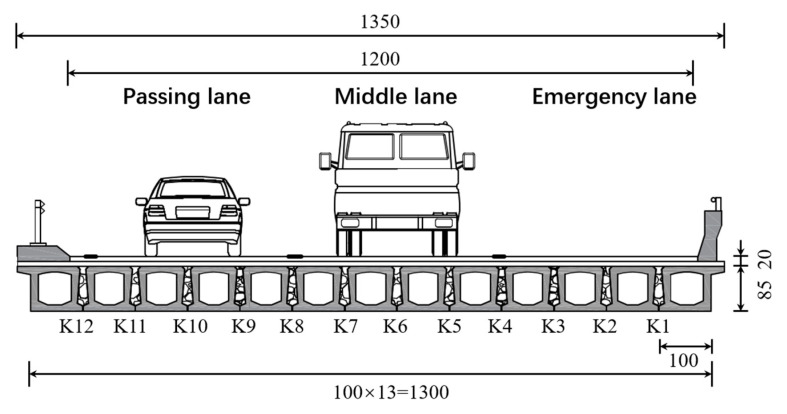
Bridge cross section (unit: cm).

**Figure 12:**
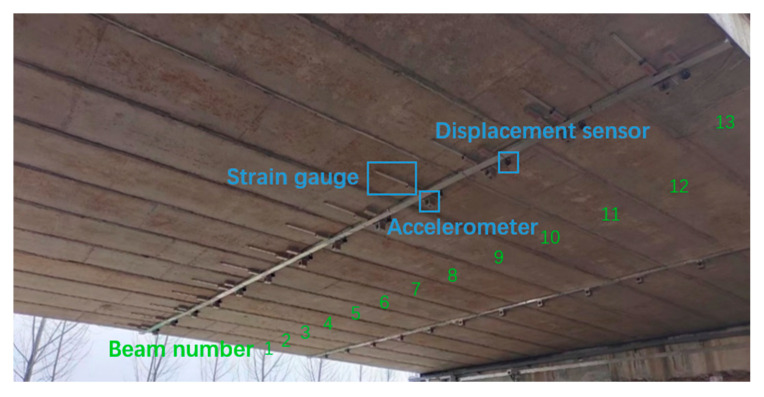
Sensor arrangement.

The method proposed in this article was applied to an actual bridge. The side view and top view of the bridge are shown in Figure 9 and Figure 10, respectively. The bridge was constructed by assembling 13 prefabricated beams with a span of 20 m. The entire bridge had eight identical spans, all simply supported girders, but the deck pavement was continuous. The thickness of the paving layer was 0.2 m. In this study, only the first span of the bridge was studied, ignoring the stiffness of the paving layer. The cross-sectional view of the bridge is shown in Figure 11. The bridge was 13.5 m wide with three lanes. The prefabricated girders had a girder height of 0.85 m and a girder width of 1 m. The acceleration sensors were installed at the bottom of each beam in the first span, as shown in Figure 12. In the following section, the notation “No. 1” is used to denote the beam number and the notation “K1” is used to denote the hinge joint number.

From Figure 10, it can be seen that there were rutting marks in the middle of the bridge, indicating that the hinge joint located in the middle may have experienced stiffness degradation. However, according to the visual inspection of the hinge joint in Figure 12, there was no significant damage to the hinge joint of the bridge, indicating that the hinge joint in the middle position may only have experienced slight degradation.

### 4.2. Data Analysis

**Figure 13:**
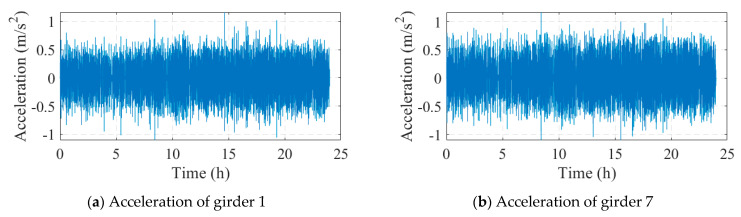
Acceleration data.

**Figure 14:**
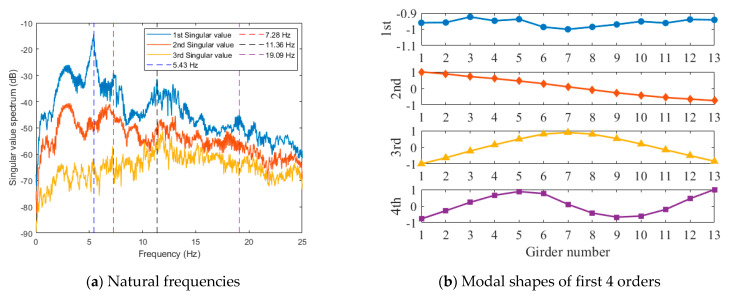
Modal identification.

A total of 13 acceleration sensors were installed at the middle span of the bridge, with a sampling frequency of 50 Hz. The maximum range of the accelerometer was ±20$\;m/s^{2}$, the sensitivity was 0.3 $V/(m/s^{2})$, and the sampling frequency range was 0.20 Hz~80 Hz. Some of the acceleration monitoring data are shown in Figure 13. The natural frequencies and corresponding mode shapes of the bridge were identified by the FDD method with the measured acceleration data, and the results are shown in Figure 14. Figure 14a is the singular value spectrum obtained by singular value decomposition (SVD) of the power spectrum matrix, from which the first four natural frequencies of the bridge could be obtained. It should be noted that according to the analysis of the vibration mode, the peak value near 3 Hz in the singular value spectrum was not the first-order frequency of the bridge but the frequency of vehicles and other loads in the traffic load. Figure 14b shows the corresponding mode shapes of the first four orders.

**Figure 15:**
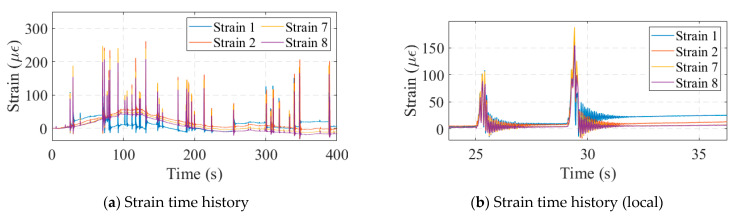
Strain data.

**Figure 16:**
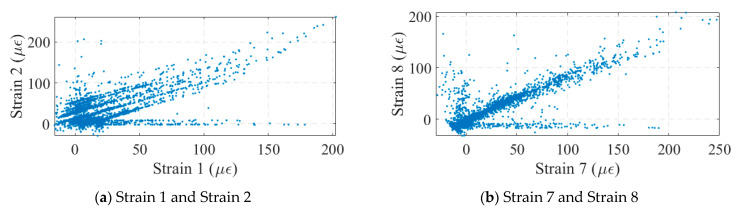
Strain correlation analysis.

Strain gauges have a measuring range of −3000 με to 3000 με and a sensitivity of 500 με/mV. The strain monitoring data for the bridge are shown in Figure 15. When vehicles passed over the bridge, the strains in each girder of the bridge changed significantly. However, the strain sensors commonly suffered from data drift, as shown in Figure 15b, as when a vehicle passed over the bridge, the strain amplitude of the bridge did not return to its initial value. This created a challenge for some of the monitoring indicators based on strain correlation. Figure 16 plots the correlation scatter plot of the neighboring strain measurement points. The drift of the No. 1 strain data was more significant, which led to a wide range of scatter distribution in the plot, as shown in Figure 16b. The distribution of many parallel lines in the graph, instead of a single line, was the result of the shift in the data from measurement point No. 1. The drift phenomenon of No. 7 and No. 8 was weaker, so the scatter points were concentrated in a narrow band range. This suggested that some strain-based monitoring indicators may have been significantly affected by data drift, which needed to be processed before being applied to real bridge monitoring.

**Figure 17:**
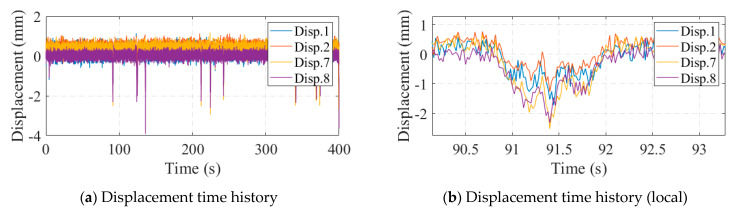
Optical displacement gauge monitoring data.

**Figure 18:**
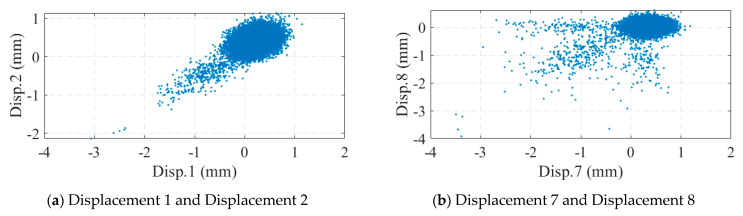
Displacement correlation analysis.

**Figure 19:**
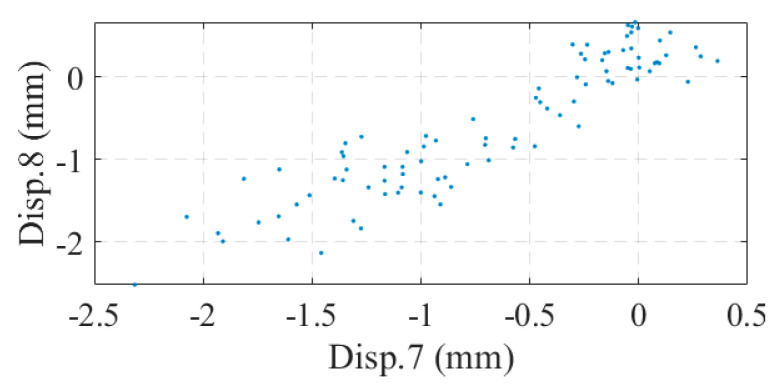
Correlation between Disp. 7 and Disp. 8 in large displacement cases.

The measuring range of the optical displacement meter was 0~600 mm and the measuring accuracy was ±0.1 mm within 30 m and ±0.2 mm within 50 m. Figure 17 illustrates the monitoring data collected from the optical displacement gauge. Compared to the strain gauges, the optical displacement gauges did not suffer from data shifting; however, there was a large amount of noise in the displacement data. When the bridge displacement was small, the displacement response was drowned out by the noise. Correlation analysis was performed on the deflection data, as shown in Figure 18. As seen in Figure 18b, the correlation between displacements No. 7 and No. 8 seemed to be very small, which contradicted the results of the strain analysis. In fact, this was caused by the noise: a large number of points in the figure were near the origin of the axes, and these points were affected by the noise. If the data with large displacement variations in Figure 17b were selected for correlation analysis, a significant correlation between displacements No. 7 and No. 8 could be found, as shown in Figure 19. This suggests that methods based on displacement correlation indicators are more susceptible to measurement noise.

### 4.3. Hinge Joint Assessment

**Figure 20:**
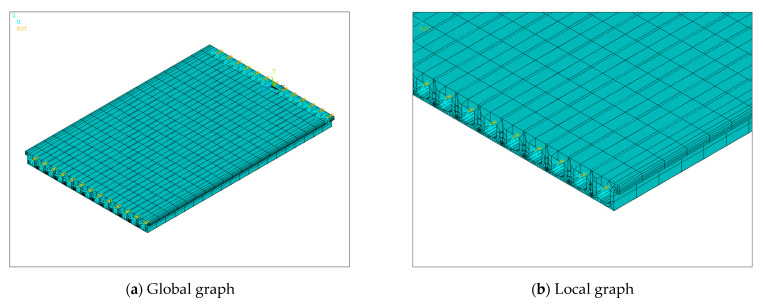
Bridge FEM. (The blue arrows indicate the direction of displacement constraints and the yellow arrows are the direction of rotation constraints).

**Figure 21:**
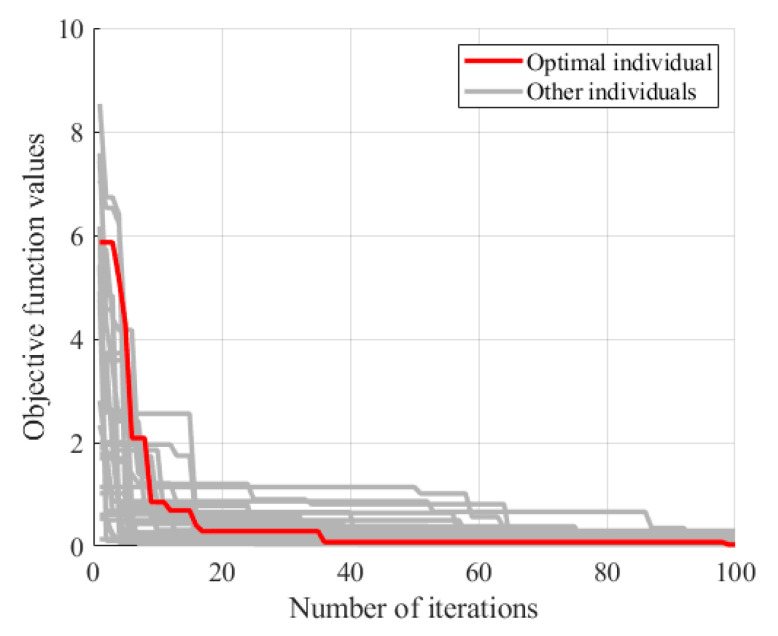
Optimization process for baseline stiffness of hinge joints.

Based on the bridge design data, an FEM of the bridge was established using ANSYS 19.2, as depicted in Figure 20. This model was simulated using the beam element BEAM188. The elastic modulus was set to $3.45\times 10^{10}\;N/m^{2}$ and the density to 2000 $kg/m^{3}$. The model consisted of 729 nodes and 716 elements. The springs between adjacent beams coupled only vertical degrees of freedom to simulate hinge joints that transmitted only shear forces. Vertical, transverse, and torsional degrees of freedom were constrained at both ends of each beam, and, in addition, the degrees of freedom along the length were constrained at one end. The beam cross-sections were accurately represented according to the design information, and the hinge joints were modeled as virtual beams. The model was then modified using the method of Section 2.2. The stiffness of the virtual beams was corrected by targeting the measured frequency of the bridge, assuming that all virtual beams had the same stiffness. The optimization process using the PSO algorithm is shown in Figure 21. The baseline stiffness obtained by the optimization algorithm was $3.65\times 10^{7}\;N/m$. It should be noted that the identified baseline stiffness was lower than the original stiffness. The damage indicators obtained from the baseline stiffness could be used to screen out hinge joints with relatively large stiffness losses.

**Figure 22:**
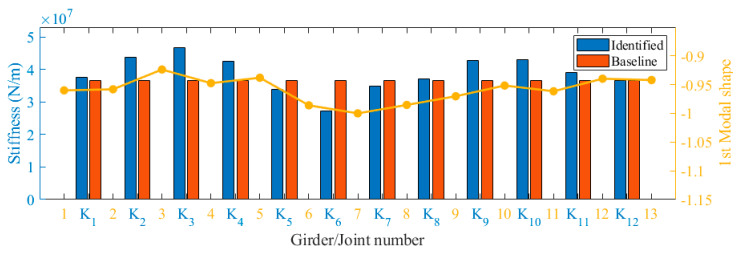
Identified results of joint stiffness.

Based on the identified frequencies and modal shapes, the stiffness of each hinge joint was determined using the method described in Section 2.1. The obtained stiffness values were then compared with the baseline stiffness, and the comparative results are presented in Figure 22. As can be seen from the figure, the identified stiffness of each hinge joint fluctuated around the baseline. The damage indicators were obtained as shown in Table 3. It was evident that hinge joint K6 exhibited the lowest stiffness, aligning with the first-order modal shape identification. The first modal shape shown in Figure 22 indicated a slight difference in the vibration amplitude of beams No. 6 and No. 7 compared to the other beams. Consequently, an inference could be drawn that potential damage might have taken place in hinge joint K6. Nonetheless, gauging the magnitude of this damage based solely on the vibration pattern index presented challenges. The proposed method not only detected the damage in hinge joint K6 but also offered a quantitative assessment of the damage extent. In addition, the hinge joint stiffness of K5 and K7 were slightly lower than the baseline values.

Overall, the results indicated that the stiffness of all hinge joints was at a high level, and no serious damage conditions occurred, aligning with the outcomes of the visual inspection. Given that hinge joint K6 was situated near the lane junction and subjected to high shear forces, the stiffness of this joint exhibited a slight degradation, which will require focused attention during future bridge maintenance to prevent significant stiffness degradation.

## 5. Conclusions

This paper introduced an innovative framework for assessing hinge joints of prefabricated AMGBs. At the core of the framework is a hinge joint stiffness estimation algorithm, eliminating the requirement for traffic interruption or load experimentation. This enables direct ascertainment of hinge joint stiffness under operational loads. Furthermore, a damage indicator relying on the stiffness ratio was proposed. The precision of this approach was validated through numerical simulation and actual bridge testing, leading to the following conclusions:

1. The numerical simulation results indicate that the proposed method for calculating the stiffness of hinge joints based on the characteristic equation of multi-beam systems can accurately identify the stiffness of hinge joints in multi-beam systems.
2. Analysis based on measured data shows that some strain correlation-based indicators may be significantly affected by data drift, while methods based on displacement correlation metrics may be susceptible to noise. In contrast, the acceleration data-based method proposed in this paper does not suffer from these problems. The assessment process does not require the closure of traffic and directly uses the monitoring data under operating conditions.
3. The actual bridge test results show that the evaluation results of the proposed method for hinge joints were basically consistent with the results of visual inspection and modal shape indicators. The method proposed in this paper can be used for the health monitoring of real bridges and to provide recommendations for the maintenance of bridges.

The proposed method directly obtains the equivalent stiffness of hinge joints, and combining this with an FEM can provide a baseline for the evaluation of bridge hinge joints. The results of the application on the real bridge show that the proposed method has practical application prospects.

## Figures and Tables

**Table 1:** Frequencies in each case (Hz).

Order	1st	2nd	3rd	4th
Undamaged	4.49	5.35	7.30	9.63
Case 1	4.49	5.20	6.93	9.34
Case 2	4.49	5.25	6.94	9.14

**Table 2:** Damage indicators of Case 1 and Case 2.

	Joint Number	Stiffness (N/m)	$D_{i}$	Joint Number	Stiffness (N/m)	$D_{i}$
Case 1	1	$1\times 10^{7}$	1.07	2	$1\times 10^{7}$	1.08
	3	$1\times 10^{7}$	1.08	4 (Damaged)	$0.4\times 10^{7}$	0.44
	5	$1\times 10^{7}$	1.07	6 (Damaged)	$0.7\times 10^{7}$	0.73
	7	$1\times 10^{7}$	1.09	8	$1\times 10^{7}$	1.10
	9	$1\times 10^{7}$	1.09	10	$1\times 10^{7}$	1.08
	11	$1\times 10^{7}$	1.08	12	$1\times 10^{7}$	1.08
Case 2	1	$1\times 10^{7}$	1.14	2	$1\times 10^{7}$	1.14
	3 (Damaged)	$0.8\times 10^{7}$	0.93	4 (Damaged)	$0.6\times 10^{7}$	0.67
	5	$1\times 10^{7}$	1.12	6	$1\times 10^{7}$	1.13
	7	$1\times 10^{7}$	1.16	8 (Damaged)	$0.8\times 10^{7}$	0.91
	9	$1\times 10^{7}$	1.14	10	$1\times 10^{7}$	1.16
	11	$1\times 10^{7}$	1.10	12 (Damaged)	$0.4\times 10^{7}$	0.56

**Table 3:** Damage indicators of real bridge.

Joint Number	Identified Stiffness (N/m)	$D_{i}$
1	$3.75\times 10^{7}$	1.03
2	$4.38\times 10^{7}$	1.20
3	$4.68\times 10^{7}$	1.28
4	$4.25\times 10^{7}$	1.16
5	$3.38\times 10^{7}$	0.93
6	$2.73\times 10^{7}$	0.75
7	$3.50\times 10^{7}$	0.96
8	$3.71\times 10^{7}$	1.02
9	$4.27\times 10^{7}$	1.17
10	$4.30\times 10^{7}$	1.18
11	$3.90\times 10^{7}$	1.07
12	$3.66\times 10^{7}$	1.00

## Data Availability

Dataset available on request from the authors. Data are contained within the article.

## References

[1] Liu S., Liu T., Zhou J., Chen L., Yang X. (2019). Relationship between shear-stress distribution and resulting acoustic-emission variation along concrete joints in prefabricated girder structures. Eng. Struct..

[2] Xia Y., Lei X., Wang P., Sun L. (2021). A data-driven approach for regional bridge condition assessment using inspection reports. Struct. Control Health Monit..

[3] Shi W., Shafei B., Liu Z., Phares B.M. (2019). Early-age performance of longitudinal bridge joints made with shrinkage-compensating cement concrete. Eng. Struct..

[4] Yuan J., Graybeal B. (2016). Asce Full-Scale Testing of Shear Key Details for Precast Concrete Box-Beam Bridges. J. Bridg. Eng..

[5] Porter S.D., Julander J.L., Halling M.W., Barr P.J. (2012). Shear Testing of Precast Bridge Deck Panel Transverse Connections. J. Perform. Constr. Facil..

[6] Sun L., Shang Z., Xia Y., Bhowmick S., Nagarajaiah S. (2020). Review of Bridge Structural Health Monitoring Aided by Big Data and Artificial Intelligence: From Condition Assessment to Damage Detection. J. Struct. Eng..

[7] Gong F., Lei X., Xia Y. (2024). Real-time damage identification of hinge joints in multi-girder bridges using recursive least squares solution of the characteristic equation. Eng. Struct..

[8] Wen X., Lei W., Dan D., Liu G. (2017). Study on a measurement index of transverse collaborative working performance of prefabricated girder bridges. Adv. Struct. Eng..

[9] Dan D., Zheng W., Xu Z. (2023). Research on monitoring index of transverse cooperative working performance of assembled multi-girder bridges based on displacement spectrum similarity measure. Structures.

[10] Zheng W., Dan D., Zhong J. (2023). Performance monitoring of assembled beam bridges using displacement spectrum similarity measure. Adv. Struct. Eng..

[11] Dan D., Xu Z., Zhang K., Yan X. (2019). Monitoring Index of Transverse Collaborative Working Performance of Assembled Beam Bridges Based on Transverse Modal Shape. Int. J. Struct. Stab. Dyn..

[12] Reiff A.J., Sanayei M., Vogel R.M. (2016). Statistical bridge damage detection using girder distribution factors. Eng. Struct..

[13] Han F., Dan D., Xu Z., Deng Z. (2022). A vibration-based approach for damage identification and monitoring of prefabricated beam bridges. Struct. Health Monit..

[14] Hu H., Wang J., Dong C.-Z., Chen J., Wang T. (2023). A hybrid method for damage detection and condition assessment of hinge joints in hollow slab bridges using physical models and vision-based measurements. Mech. Syst. Signal Process..

[15] Dan D., Zhao Y., Wen X., Jia P. (2018). Evaluation of lateral cooperative working performance of assembled beam bridge based on the index of strain correlation coefficient. Adv. Struct. Eng..

[16] Xu Z., Dan D., Deng L. (2021). Vibration-Based Monitoring for Transverse Cooperative Working Performance of Assembled Concrete Multi-Girder Bridge: System Design, Implementation and Preliminary Application. Int. J. Struct. Stab. Dyn..

[17] Xia Q., Zhou Y.-C., Cheng Y.-Y., Zhang J. (2023). Hinge Joints Performance Assessment of a PC Hollow Slab Bridge Based on Impact Vibration Testing. Struct. Control Health Monit..

[18] Walsh K.K., Kelly B.T., Steinberg E.P. (2014). Damage Identification for Prestressed Adjacent Box-Beam Bridges. Adv. Civ. Eng..

[19] Abedin M., Basalo F.J.D.C.Y., Kiani N., Mehrabi A.B., Nanni A. (2022). Bridge load testing and damage evaluation using model updating method. Eng. Struct..

[20] Zhan J., Zhang F., Siahkouhi M., Kong X., Xia H. (2021). A damage identification method for connections of adjacent box-beam bridges using vehicle–bridge interaction analysis and model updating. Eng. Struct..

[21] Kelly S.G., Srinivas S. (2009). Free vibrations of elastically connected stretched beams. J. Sound Vib..

[22] Di F., Sun L., Chen L. (2021). Optimization of hybrid cable networks with dampers and cross-ties for vibration control via multi-objective genetic algorithm. Mech. Syst. Signal Process..

[23] Whitley D. (1994). A genetic algorithm tutorial. Stat. Comput..

[24] Bansal J.C., Bansal J.C., Singh P.K., Pal N.R. (2019). Particle Swarm Optimization. Evolutionary and Swarm Intelligence Algorithms.

[25] Kameyama K. (2009). Particle Swarm Optimization—A Survey. IEICE Trans. Inf. Syst..

[26] Van Overschee P., De Moor B. (1993). Subspace algorithms for the stochastic identification problem. Automatica.

[27] Pappa R.S., Elliott K.B., Schenk A. (1993). Consistent-mode indicator for the eigensystem realization algorithm. J. Guid. Control Dyn..

[28] Brincker R., Zhang L., Andersen P. (2000). Modal identification from ambient responses using frequency domain decomposition. Proceedings of the IMAC-XVIII: A Conference on Structural Dynamics ‘Computational Challenges in Structural Dynamics’.

